# Mixed methods developmental evaluation of the CHOICE program: a relationship-centred mealtime intervention for long-term care

**DOI:** 10.1186/s12877-018-0964-3

**Published:** 2018-11-13

**Authors:** Sarah Wu, Jill M. Morrison, Hilary Dunn-Ridgeway, Vanessa Vucea, Sabrina Iuglio, Heather Keller

**Affiliations:** 1 University of Waterloo, 200 University Avenue West, Waterloo, Ontario N2L 3G1 Canada; 2 Research Institute for Aging, 250 Laurelwood Drive, Waterloo, Ontario N2J 0E2 Canada

**Keywords:** Dining, Complex intervention implementation, Evaluation, Program development, Implementation science, Mealtimes, Long-term care, Residential care, Relationship-centred care, Personal support workers

## Abstract

**Background:**

Mealtimes are important to quality of life for residents in long-term care (LTC). CHOICE (which stands for Connecting, Honouring dignity, Offering support, supporting Identity, Creating opportunities, and Enjoyment) is a multi-component intervention to improve relationship-centred care (RCC) and overall mealtime experience for residents. The objective of this developmental evaluation was to determine: a) if the dining experience (e.g. physical, social and RCC practices) could be modified with the CHOICE Program, and b) how program components needed to be adapted and/or if new components were required.

**Methods:**

A mixed methods study conducted between April–November 2016 included two home areas (64 residents; 25 care staff/home management) within a single LTC home in Ontario. Mealtime Scan (MTS), which measures mealtime experience at the level of the dining room, was used to evaluate the effectiveness of CHOICE implementation at four time points. Change in physical, social, RCC dining environment ratings and overall quality of the mealtime experience over time was determined with linear mixed-effects analyses (i.e., repeated measures). Semi-structured interviews (*n* = 9) were conducted with home staff to identify what components of the intervention worked well and what improvements could be made.

**Results:**

Physical and overall mealtime environment ratings showed improvement over time in both areas; one home area also improved social ratings (*p* < 0.05). Interviews revealed in-depth insights into the program and implementation process: i) Knowing the context and culture to meet staff and resident needs; ii) Getting everyone on board, including management; iii) Keeping communication lines open throughout the process; iv) Sharing responsibility and accountability for mealtime goals and challenges; v) Empowering and supporting staff’s creative mealtime initiatives.

**Conclusions:**

This developmental evaluation demonstrated the potential value of CHOICE. Findings suggest a need to: extend the time to tailor program components; empower home staff in change management; and provide increased coaching.

**Electronic supplementary material:**

The online version of this article (10.1186/s12877-018-0964-3) contains supplementary material, which is available to authorized users.

## Background

Mealtimes have the potential to foster and support important and meaningful social relationships over the life course [1, 2]. Consequently, food and mealtimes are considered key aspects of quality of life and satisfaction for residents living in long-term care (LTC) homes [3–5]. Specifically, mealtimes in LTC have the capacity to act as a starting point for creating and sustaining social relationships [6]. The concept of reciprocity is strongly associated with meals [1, 7]; to extend this idea to LTC environments is to arrive at what many refer to as relationship-centred care (RCC), a social model that reflects the “importance of interactions amongst people as the foundation of any therapeutic or healing activity” [8]. The RCC model was purposefully selected for this intervention over the more commonly used philosophy of person-centred care, as it actively situates the resident within their social networks instead of viewing the resident as an unassociated, independent being [9]. Further, we may more effectively position the resident within the context of important and significant relationships (i.e., care staff, dietary aids, home management, etc.) during mealtimes in LTC through the adoption of an RCC model of care [9, 10].

A recent study identifying the determinants of poor food intake among LTC residents found that the mealtime experience and the way care is provided at meals plays a critical role in supporting food intake [11]. Yet, only a handful of intervention studies have attempted to improve the mealtime experience in LTC by intentionally or unintentionally including a psychosocial component in their design [12, 13]. Interventions developed to improve social interaction among residents have focused on the built dining environment, such as smaller dining spaces [14] while others have created meaningful mealtime opportunities for residents through involving them in meal preparation activities, such as making breakfast [15]. Rather than emphasizing the social aspects of routine meals, care staff or volunteer training interventions focus on tasks to promote food intake [12, 13], and no program identified to date has explicitly promoted dignity, honouring of individual identity, or the supporting of social connections between residents and staff at meals. Specifically, no training described in prior research has adopted an RCC model of care as a basis from which to enhance team member (i.e., care staff) capabilities or attempt to change behaviour to enhance reciprocal dining experiences for LTC residents.

### Development of the CHOICE program

The concept of the CHOICE Program was developed over several years of research that identified the potential for dining in LTC to be more relationship-focused. Early research by this team identified the role meals played in the family home for persons living with dementia and their family care partners [16]. As families moved into residential environments, the understanding that these meals did not live up to expectations was identified [17]. Observations in a variety of residential environments demonstrated the social engagement and potential for building of relationships for residents and staff [6] and finally the concept of relational dining which encapsulates RCC and person-centred care practices was formed [18]. It was believed that the overall experience of a mealtime that captured physical, social and relationship-centred components could be measured and thus the Mealtime Scan (MTS) was developed and used in a large multi-site study [19]. The way that care was provided, and specifically more person- and relationship-centred was found to be associated with improved food intake [19]. This team then embarked on a journey to develop the CHOICE Program based on this acquired knowledge and experience with mealtimes in LTC settings.

The first step in development of the training program was to articulate more fully what relationship-centred and relational care could look like in the dining room and draw upon important principles from a substantive theory developed in a previous study with persons living with dementia and their care partners on the meaning of mealtimes [17]. This lead to the development of the CHOICE Principles which stand for Connecting, Honouring dignity, Offering support, supporting Identity, Creating opportunities and Enjoyment (Table 1). These principles were discussed at a variety of provider conferences and decision-maker discussion groups to see whether they resonated with those outside of the research program. In line with RCC, the program needed to enhance the mealtime experiences for residents and team members (i.e., Personal Support Workers and Dietary Aids) by changing team member attitudes, knowledge, and behaviour in order to promote and re-emphasize the importance of meaningful social interactions with residents. To be effective and responsive to the changing needs of residents and team members, the CHOICE Program needed to be tailored to the individual home area based on the priorities identified by the team members. Thus, the focus of training was on the principles and how they could be enacted within a specific care setting. Training materials for the CHOICE Program were developed based on models typically utilized by LTC home chains, and where this developmental evaluation was to be conducted. This included: a) a presentation that described each CHOICE Principle, b) scenarios on each principle for staff huddles (i.e., brief direct care staff meetings), c) brainstorming ideas for making specific improvements in physical, social and relationship-centred care, and d) verbal and visual reminders to enact identified ideas for mealtime improvements. The physical dining room and the team members within it were the targets for the intervention.

**Table 1:** The Six Principles of CHOICE

Connecting	Feeling a sense of togetherness and belonging with others. It is important to get to know who the resident is and how they like to connect with others at mealtimes.
Honouring Dignity	Respecting a resident’s decisions, choices, and actions at mealtimes.
Offering Support	Adapting to what a resident needs in the moment. The amount and type of support may change from meal to meal. It is best to ask a resident what they need or want instead of making assumptions.
Supporting Identity	Accepting and acknowledging a resident for who they are today, while working to understand their life story that includes significant events, roles, and important relationships. Who they are will impact how they experience mealtimes.
Creating Opportunities	Engaging in meaningful mealtime roles is important to all of us. Involve residents in familiar or new meal related activities to make them feel a part of a familiar routine.
Enjoyment	Creating a welcoming, relaxed, and friendly dining environment can lead to more enjoyment at mealtimes. Create mealtime events for special occasions, such as birthdays or cultural holidays.

## Methods

### Study aim

This study describes the developmental evaluation of the CHOICE Program for LTC staff delivered in a single pilot site. The aim of this study is to determine: a) if the mealtime experience (e.g. physical, social and RCC practices) could be modified with the CHOICE Program, and b) how program components needed to be adapted and/or if new components were required.

### Research design

Developmental evaluation is used in the early stages of an intervention or program to understand what works and what does not, especially in multi-component programs designed for complex environments [20]. A key purpose of such an evaluation is to learn and adapt the intervention as it is being delivered, and thus a ‘bottom-up’ approach is needed [20]. This form of evaluation is especially relevant for complex interventions that involve coordination of diverse staff and attempts to change their social interactions with others. As a result, components of CHOICE were revised or discarded and new components developed during the course of this evaluation. An explanatory sequential mixed methods (QUANTITATIVE ➔ qualitative) pre-test post-test time series design was used to evaluate the CHOICE Program. This evaluation began with the collection of quantitative data using the observational tool, the Mealtime Scan for Long-Term Care (MTS), at four time points during a 24-week period. Quantitative results informed the subsequent qualitative data collection using semi-structured interviews [21]. Findings were merged to address study objectives. Ethical clearance was obtained from the University of Waterloo Office of Research Ethics (#21413).

### Intervention participants and setting

It was anticipated that learnings would be enhanced and strengthened if two home areas (sometimes referred to as "care units") - Parker and Wellesley (pseudonyms) - in a single LTC were included, as resident differences with respect to eating capacity would likely influence types of team member activities undertaken to make improvements. The LTC home was located in Southern Ontario, Canada, and is part of an Ontario chain of for-profit homes. The Schlegel-University of Waterloo Research Institute for Aging (RIA) facilitated the incubation of this innovation in a research-friendly home so that the learnings from this work could contribute to the acceleration and eventual mobilization of the innovation to the broader LTC sector. The participating home has a total of 192 beds within 6 home areas and was also selected based on a self-identified need to refocus team member efforts on resident mealtimes. As well, this home had minimal prior exposure to external mealtime-focused research initiatives. As the quantitative data collection procedure was based on dining room-level observations, written consent was not required as per the University of Waterloo Office of Research Ethics from individual team members and residents, as only global aspects of the mealtime experience were being assessed. Informational posters and newsletters were provided to residents, families, and team members prior to the start of implementation to ensure that everyone who lived and worked in this home area was aware of the initiative and the use of observations to measure potential changes. Signs were posted advising the home areas when meal observations were to occur. Those care team members and home management who played critical roles in the implementation of the CHOICE Program were invited to participate in interviews in the qualitative phase; informed written consent was provided.

### Implementation overview

Prior to pilot implementation, the research team (i.e., Principal Investigator HK, HD Study Coordinator, and doctoral student, SW) met with the home’s administrators to discuss program components, implementation process, and potential barriers and facilitators of care team behaviour change during mealtimes (Table 2). A mutual agreement was reached on study timeline (i.e., 8-month involvement of care staff implementation process and evaluation), time commitments (i.e., time dedicated to staff huddles and coaching phone calls), support resources (i.e., informational resources on care delivery, program print materials), and outcome goals (i.e., what was feasible for each home area to achieve in this program). The home’s administrators identified two Mealtime Champions (MT Champions) per home area to act as leaders to guide fellow team members through the implementation process. MT Champions play a critical role as point of contact for the interventionists as a way to give and receive feedback during implementation. The MT Champions were selected based on: a) their interest and dedication to care improvement, b) their positive social influence among their colleagues and reciprocated respect they received, and c) full-time employment to ensure their knowledge of the home area. Each home area attempted to work on one CHOICE Principle at a time to raise awareness and identify areas for improvement. Flexibility in the program allowed home areas to spend as much time as needed on a principle. The Director of Food Services (DFS), acted as both an internal resource and CHOICE advocate to hold the team members within each home area accountable to their goals each week. It became apparent immediately that the MT Champions and DFS needed further support post training to stimulate change among the care team. The research team took on this role collectively; the PI provided support on techniques and theory to make change; the study coordinator who was an employee of the RIA facilitated communications among home management, the dining team and the researchers, while the doctoral student visited the site, initially with the study coordinator, and took on the role of ‘CHOICE Coach’ on-site as the implementation progressed. The Coach visited the study site twice per month for 5 months, spending 1 day in each home area to attend team member huddles, support the completion of the huddle diary, observe mealtimes, gain feedback from team members and residents, and strategize and problem-solve with the MT Champions. She also took this time to meet with the DFS to review progress reports generated by the research team using MTS results in order to strategize on ways to support team members in areas identified as needing improvement. Over the last 3 months of implementation, the Coach reduced in-person presence in order to promote capacity and independence among the MT Champions and team members; however, bi-weekly coaching teleconference meetings continued with the research team.

**Table 2:** CHOICE Intervention Components and Functions

Intervention Components	Description	Dosage	Frequency over 8 months
Education Session and Training Modules	Overview of program components and best-practices to enhance mealtime experience for residents using relationship-centred approaches. Education session was developed into training module to ensure all staff received education sessions.	45 min./home area	1 per home area or as needed for new hires/refresher for current team members
Staff Huddles and Huddle Diary	Mealtime-focused huddles scheduled during shift changeover to promote CHOICE Principles, problem-solve, record progress in huddle diaries, and facilitate communication between care staff.	5–10 min./huddle	1 x week or as needed
Visual Reminders	Posters provided weekly reminders of each CHOICE Principle for care staff and posted strategically around dining room and servery.	1 poster/week	2–3 posters per dining room or as needed
Reference Binder	CHOICE reference binder provided resources staff would need to carry out program, including program overview, huddle schedule, huddle diary sheets, and reminder posters.	1 binder/home area	As needed by care staff and leadership
MT Champion Meetings	Teleconference meetings held when CHOICE Coach not on site in order to discuss progress, identify areas for improvement, problem-solve, and identify ways to respond to MTS feedback. In-person meetings held when Coach on site.	10–20 min/home area	1 per week
Continuous Feedback	Progress reports were generated based on MTS data collected by external auditors. Reports were reviewed by the MT Champions, DFS, project coordinator, with the research team prior to being shared with the care staff and other home administrators. Reports assisted in identifying areas that had improved and/or needed improvement	Comprehensive report based on MTS Data.	Baseline, 8 weeks, 16 weeks, 28 weeks for each home area (missing 5th meal audit because of outbreak)
CHOICE Coach	CHOICE Coach worked closely with MT Champions and DFS to facilitate and support each component of CHOICE program and assist in tailoring components when needed. Coach provided feedback on barriers and facilitators of program components, implementation process, and important contextual factors, such as organizational climate.	In-person visit: 5–7 h per home area. MT Champion Meetings 10–20 min.	In-person visit 2 per month for 5 months for each home area. Teleconference 1 per week for 3 months.

Abbreviations: *MT* mealtime, *MTS* Mealtime Scan, *DFS* director of food service

As the MT Champions and the DFS started to consider how to make improvements, it was recognized that they needed coaching on change management. The Theoretical Domains Framework was chosen as a basis for coaching on strategies to make change [22, 23]. In this framework, fourteen domains (i.e., knowledge, skill, beliefs about capabilities, intentions, goals, etc.) can be condensed into three core components in relation to behaviour change: capability, opportunity, and motivation (e.g. COM-B; [24]). The COM-B model was selected specifically for this LTC intervention as it places emphasis on the importance of context (i.e., opportunity) when looking to change behaviour, which is critical when designing feasible and acceptable interventions for health care environments. Without explicitly educating team members on this framework, coaching was provided by the research team to support the adoption, uptake and execution of the program components. For example, celebrating success was an incentive to further motivate team members to complete a specific care activity.

The intervention evolved over the course of the pilot, as is typical of developmental evaluation [20]. It ultimately included 8 components that supported team members in their change efforts, as outlined in Table 2. Team members and the research team worked to tailor these components over the course of the implementation process to meet specific needs of the individual home areas. For example, the in-person educational session on CHOICE Principles was translated into an online education module and made available on the home’s intranet training platform for those team members and administrators who were not present for the in-person sessions, as well as new team member hires.

### Quantitative phase

#### Data collection

To understand impact of CHOICE on mealtime processes and interactions among residents and team members, several mealtime observations were conducted every two months using a standardized tool. Mealtime audits were completed with the Mealtime Scan [MTS] [25]. This observational tool is a construct valid and reliable assessment that measures the psychosocial environment, as well as physical aspects of a dining environment that impact the mealtime experience [25, 26]. Observations are conducted at the dining room level, and not at the individual resident level, to attain an overall impression and summary of key aspects of the observed meal. As part of this developmental evaluation, a modified version of MTS was trialed to determine its responsiveness to the intervention. Modifications as recommended after extensive use [11, 25] included more detailed tracking of social interactions and improved scaling of items (i.e. dichotomous items changed to a 0–4 scale) that are intended to promote responsiveness to change over time. The physical environment meter (e.g. temperature, sound, humidity, and illumination) was removed from the original tool, as the protocol to collect these data was potentially disruptive to mealtimes [25]. In addition to the physical, social and person/relationship-centred summative scales included on the original MTS, a summative scale was also created to capture the overall quality of the dining environment; preliminary data demonstrated good inter-rater reliability of this summative scale (intraclass correlation coefficient = 0.76; unpublished). Maximum score for these four MTS summative scales was 8. Further details on the MTS are described in Keller et al., 2018 [25].

Two trained assessors arrived in their respective dining room several minutes before the scheduled meal start time, before residents entered, and continued observation until the end of the meal when most residents had left the dining room. Data collection was originally planned to be carried out at baseline, 8 weeks, 16 weeks, 24 weeks, and 32 weeks, however, due to concerns about illness outbreak and other home events (i.e., special events, accreditation, etc.), data collection could not be carried out at Week 32. At each of the four time points, the MTS was completed at 5 meals (1 breakfast, 2 lunches, 2 dinners) over a two-day period. Five meals had been previously used to assess the mealtime environment [25] in a large multi-site study and found to represent the meal context. The same assessor completed all assessments (*n* = 20) in the same assigned home area throughout the study to promote consistency.

Resident-level data were collected from the inter-RAI Minimum Data Set (MDS) to characterize those living in each area. Data included residents’ age, sex, Cognitive Performance Score (CPS), and Activities of Daily Life – Long Form (ADL-LF). CPS is an ordinal scale from 0 to 6 that rates residents on their cognitive capacity, where 0 = intact condition and 6 = high cognitive impairment [27]. For this analysis, CPS score was dichotomized into two groups: CPS < 3 and CPS ≥ 3 to differentiate between distinct levels of cognitive capacity. The ADL-LF is a 7-item summary scale that measures a resident’s capacity to perform activities of daily living (i.e., transfers, toilet use, and eating). Higher values on the continuous score (maximum = 28) indicate more impairment on ADLs [28].

#### Data analysis

To provide context, descriptive comparisons were made between home areas based on resident data and mealtime characteristics that were not expected to change with the intervention (e.g., number of residents, staff) using chi-square or two-sample t-tests (or non-parametric equivalent, e.g., Wilcoxon signed rank test). Each of the four MTS summative scales and subscales (e.g. Mealtime Relational Care Checklist) were tested for normality and described (mean, standard deviation [SD]) by time point and dining room. Linear mixed models (Proc MIXED) with repeated measures over time within dining room were used to analyze each of the four MTS summative scales as outcomes. Dining room, time point (categorical with time 0 as referent), and the interaction between dining room and time point were included in the model as independent variables tested as fixed effects. As both home areas received the CHOICE program, there was no control. The time effect demonstrated the potential for the MTS scales to change over time with implementation of the program, while time-home area interactions demonstrate differences by home area in this effect. Data were analyzed using SAS® Studio Statistical Software (SAS Institute Inc., Cary, North Carolina). Statistical significance was determined at *p* < 0.05 unless otherwise specified.

### Qualitative phase

#### Data collection

Semi-structured interviews were used to explain quantitative results rather than confirm. Specifically, interviews identified what program components worked well (e.g. using data to stimulate change, educational activities), the implementation process (e.g. use of champions to lead effort), especially as it varied among home areas. Key informants were recruited based on their involvement with the implementation of the CHOICE Program (i.e., MT Champions, management, highly involved team members) at the completion of the study. Interview guides for team members and home management were developed based on key findings from the progress reports generated for each home area (i.e., MTS data over time). In particular, the following areas were addressed during the interviews:

- *Team members:* their perceptions of the impact of the intervention on resident mealtime experience; their experiences adopting and carrying out intervention components; their opinions on how the program could be improved and sustained; what mealtime changes were noticed
- *Home Management:* their perceptions of the program components and how they were received by team members; their experiences with the implementation process; how better to support team members to sustain the program; what mealtime changes were noticed.

In addition, brief qualitative comments were recorded by each of the auditors on each MTS audit form to account for and contextualize unusual events that may have impacted a particular meal’s score during MTS observations. These were reviewed and used to support the linkage between quantitative and qualitative findings. Interviews were conducted by the CHOICE Coach (SW) over one month following the end of the intervention in November 2016.

#### Data analysis

Data analyses were conducted concurrently with data collection conducted by SW until informational redundancy was reached [29]. Interviews were transcribed verbatim and were then analyzed using a generalized inductive approach, whereby codes were applied to small segments of data and individual codes collapsed into larger categories that were interpreted into themes [30]. Emergent codes and the formation of categories and overarching themes were reviewed and discussed with co-authors HK, VV, and SI throughout analysis. The analytical process focused on the intervention implementation, the intervention components, and the changes that occurred during mealtimes on each home area. MTS text data was coded deductively and incorporated into the main interview themes in order to contextualize findings to the dining areas [31].

## Results

A total of 64 residents resided in the two home areas that participated in the study (Wellesley *n* = 32; Parker *n* = 32). Descriptive characteristics of residents are presented in Table 3. The average age of residents from both home areas was 85 years, with the majority of residents being female (70%). Data from the Minimum Data Set (MDS, [28]) collected during the baseline period indicates that 65% of residents living in Wellesley had a Cognitive Performance Scale (CPS) Score of 3 or greater, as compared to Parker where less than half of residents showed signs of significant cognitive impairment (CPS ≥ 3); this difference was not statistically significant. Residents in the two home areas did not differ on mean ADL-LF scores. However, the observations from the MTS indicated that Wellesley had a significantly greater number of residents who required assistance with tasks related to eating (mean = 4.8 ± 0.9 persons/meal) than Parker (mean = 3.8 ± 1.2; *p* < 0.01).

**Table 3:** Resident Characteristics of Two Home Areas (*N* = 64)

Characteristic	Both Home Areas (*N* = 64)	Wellesley (*n* = 32)	Parker (*n* = 32)
Age, mean (SD)	85 (11.7)	84 (13.3)	85 (10.0)
Male (n)	30.4% (4)	26.1% (6)	34.8% (8)
CPS Score,^a^ mean (SD)	2.6 (1.7)	3.1 (1.5)	2.0 (1.7)
Moderate/severe impairment (n)	52.2% (24)	65.2% (15)	39.1% (9)
ADL-LF^b^ Score, mean (SD)	16.4 (8.7)	16.9 (9.3)	16.0 (8.3)

^a^*CPS* Cognitive Performance Scale, scoring 0–6; scores ≥3 indicate moderate/severe cognitive impairment

^b^*ADL-LF* Activities of Daily Living Long Form Scale, scoring 0–28 with higher scores indicating more impairment of ADL independence performance

Note: Home areas were not significantly different on any of the resident characteristics, *p* < 0.05

A total of 16 team members (10 Personal Support Workers (PSW), 3 Dietary Aids (DA), 2 Registered Practical Nurse (RPN), 1 Recreational Therapist) and 5 members of home management (2 home area coordinators, 1 DFS, 1 Assistant DFS, 1 Director of Care, 1 quality indicators manager) received CHOICE training. Four PSWs, two from each home area, were selected as MT Champions by home management. However, half way through the implementation process only one Champion remained on each unit due to staff scheduling changes.

### Quantitative phase

#### Descriptive characteristics of two dining areas assessed by MTS

The two dining areas were statistically different in key physical characteristics over the course of the intervention (Table 4); Parker had more residents eating in the dining room than Wellesley (*t* = − 5.86, *p* < 0.0001). The total number of team members in either dining room did not significantly differ. However, in terms of team members’ mealtime activities, a statistical significant difference was found between the number of team members passing food (Wilcoxon; z = − 4.68, *p* < 0.0001) as well as the number of team members assisting residents with their meals (Wilcoxon; z = 4.06, *p* < 0.0001) with Wellesley having fewer food servers, but more team members assisting with eating. There was no observed difference between the number of family/volunteers present during meals, nor the number of residents eating outside the main dining area.

**Table 4:** Descriptive Characteristics of Two Dining Areas Assessed by MTS Over All Observed Meals (*n* = 40)

Variable	Both Home Areas	Wellesley	Parker
Residents in dining room, mean (SD)	24.3 (2.90)	22.4 (1.98)	26.3 (2.27)*
Any staff who entered dining room during meal observation, mean (SD)	6.7 (1.38)	7.2 (1.42)	6.3 (1.23)
Staff serving food^a^, mean (SD)	2.1 (0.94)	1.4 (0.51)	2.8 (0.77)*
Staff assisting residents to eat^a^, mean (SD)	2.6 (0.98)	3.2 (0.70)	2.0 (0.82)*
Family/Volunteers, mean (SD)	0.6 (0.89)	0.4 (0.59)	1.0 (1.05)
% of meals where at least 1 resident eating meal in adjacent area, % (freq.)	55.0 (22)	63.6 (14)	36.4 (8)

*Difference between home areas is statistically significant, *p* < 0.001

^a^Staff involved in meal service included full-time and part-time staff, LPN, and DA

Abbreviations: *MTS* Mealtime scan, *SD* Standard Deviation

#### MTS changes over time by home area

Five meals were observed at each of four time-points in each dining room, for a total of 20 mealtime observations per home area. Linear mixed models were used to determine change in MTS summative scales (i.e., physical environment, social environment, and overall quality of mealtime environment) over time for both home areas. Table 5 presents the descriptive data for each of the summative scales at each time point by home area. After adjusting for repeated measures across time within each dining room, the physical (*p* < 0.01), social (*p* = 0.02), and overall quality (*p* = 0.02) of the dining environment were significantly different, with improvements in Parker for all three scales, while Wellesley did not experience an improvement in the social summative scale. Of the MTS subscales there were several differences between dining rooms, however, only the Mealtime Relational Care Checklist score showed a significant improvement over time (*p* < 0.01) (Additional file 1: Table S1) [26].

**Table 5:** Descriptive and Linear Mixed Model Analysis of MTS Summative Global Scores At Each Time Point By Home Area

Time Point (weeks)	Descriptives by dining room		Mixed model analysis with interaction	
	Summative Scale Scores, Mean (SD)^a^		Effect	*p*-value^b^
	Wellesley	Parker		
	Physical Environment		Physical Environment	
0	5.2 (0.84)	4.8 (0.45)	Dining Room	0.37
8	5.6 (0.89)	4.6 (0.89)	Time	< 0.01
16	4.6 (0.55)	5.2 (0.45)	Dining Room x Time	0.09
24	6.2 (0.84)	6.2 (0.45)		
	Social Environment		Social Environment	
0	5.4 (1.14)	4.4 (0.55)	Dining Room	0.04
8	4.4 (0.55)	4.6 (0.89)	Time	0.02
16	3.0 (1.00)	5.6 (1.14)	Dining Room x Time	< 0.01
24	5.2 (1.30)	6.0 (0.71)		
	Relationship-Centred Care		Relationship-Centred Care	
0	4.4 (1.14)	4.2 (0.45)	Dining Room	0.15
8	5.0 (0.71)	4.8 (0.84)	Time	0.40
16	3.8 (1.30)	5.6 (0.55)	Dining Room x Time	0.09
24	4.8 (1.30)	5.2 (1.10)		
	Overall Quality of Dining Environment		Overall Quality of Dining Environment	
0	4.8 (0.84)	4.2 (0.45)	Dining Room	0.41
8	5.0 (0.71)	4.6 (0.89)	Time	0.02
16	4.2 (0.84)	5.6 (0.55)	Dining Room x Time	0.02
24	5.4 (0.89)	5.8 (0.84)		

^a^*n* = 5 observations per time point per dining room

^b^*P*-values from type 3 test of fixed effects

**Fig. 1:**
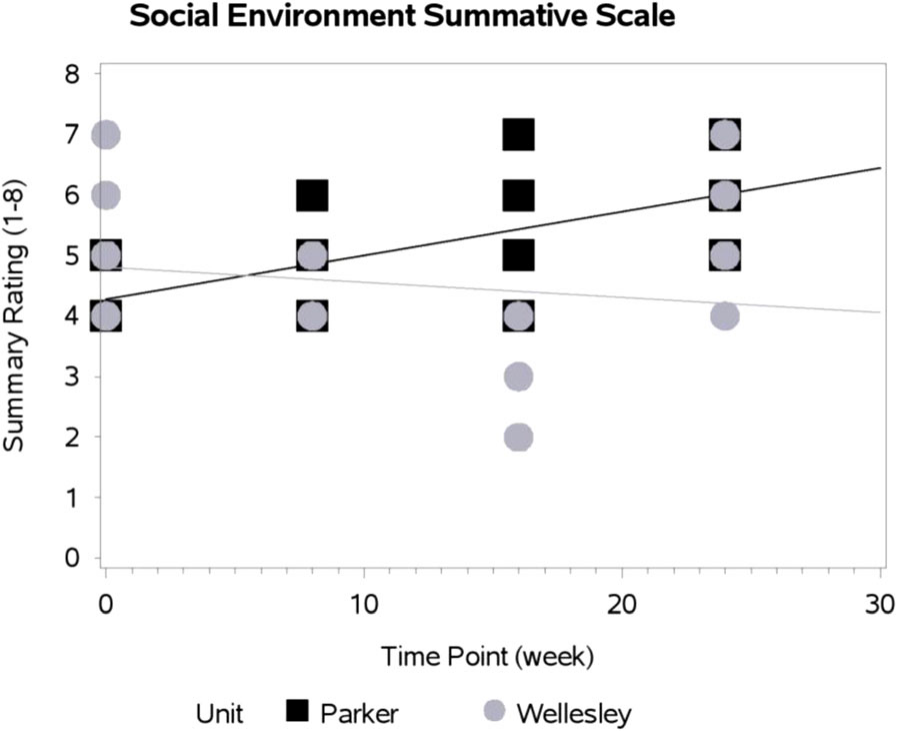
Change In Social Environment Summative Scale By Home Area Over Time

**Fig. 2:**
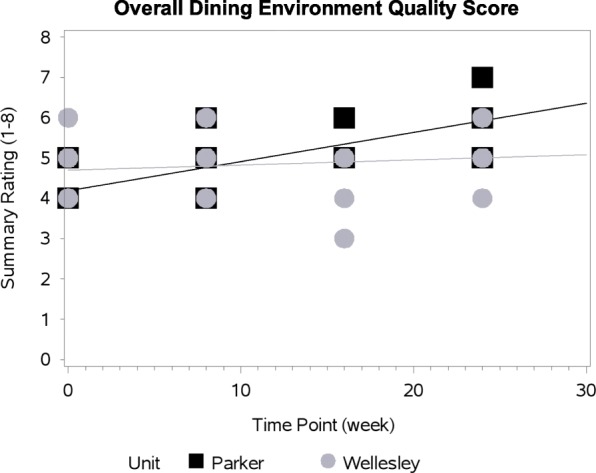
Overall Quality Of Dining Environment Summative Scale By Home Area Over Time

Models of the social environment and overall quality scores demonstrated significant interactions between home area and time. Significant interactions indicate that changes in these MTS scores across the time points varied by dining room; the intervention’s effect was not consistent over time for the two home areas. Figures 1 and 2 display the difference in change from baseline of social environment score and the overall mealtime environment rating between Wellesley and Parker. The social environment scores slightly decreased in Wellesley over the four observation points, whereas Parker’s social environment improved (Figure 1; Table 5). Overall quality of dining environment improved for Parker, however, Wellesley had a more modest improvement maintained for this scale from baseline (Figure 2; Table 5).

### Qualitative phase

Nine interviews were conducted with four PSWs (two of whom were MT Champions), two DAs, two home management members (HM), and one registered nurse (RN). Six themes emerged from the analysis. In addition, we provide key learnings and/or materials developed in response to the needs of each dining room identified during the implementation process for each theme (Table 6).

**Table 6:** Learnings and Improvements to the CHOICE Program

Findings	Learnings	Planned Improvements to Program
New Appreciation for Mealtimes: “It’s not about us; it’s about them.”	• CHOICE provided dedicated time for care staff and management to reflect and reconsider the meaning of mealtimes within their home area. • Care staff took advantage of this opportunity to share insights, learnings, and revelations amongst care teams as another way to reinforce the importance of what they are undertaking. • Staff huddles and meetings serve as a great opportunity to engage in self-reflection.	• Staff huddles and meetings proved to be more beneficial to staff to communicate with one another than using Huddle Diaries. The use of diaries ceased, which left additional time for staff to consider what aspect of mealtimes were improving, and what needed more attention. • Self-reflection based on checklists of relationship-centred care practices at mealtimes. • Adapting training on CHOICE Principles to video to allow for individual review and goal setting.
Knowing context and culture	• Additional time is needed prior to implementation to tailor the intervention components to each home area, support communication across all stakeholders and build consensus on what needs to be improved. • Changes to both the physical and social environment can improve the mealtime experience. Taking time to reflect on both environments and how they interact with one another is important. • Spending time with staff discussing how each CHOICE Principle would be enacted during their mealtimes and collectively identifying specific approaches to make change as opposed to offering vague recommendations that are not relevant to their context.	• Program extended to 52 weeks to allow for a preparatory phase where a CHOICE Dining Team is developed and trained on change management techniques. • Engagement of the residents, family and greater team with baseline findings and what areas need to be improved and identification of priority areas for change based on the perspectives of all stakeholders • Self-assessment checklist for mealtime practices as well as physical aspects of dining. • CHOICE Dining Team takes priority areas identified by family, residents and the greater care team and develops concrete action plans that are negotiated and communicated with all stakeholders in the area.
Getting everyone on board	• Additional time spent at the early stages of implementation to ensure that everyone understands what changes need to be made is critical to buy-in from care staff and management. • The development of a dining team that includes members from home management would assist in tailoring implementation components that were acceptable and feasible for a specific home area • Basic change management principles should become part of educational component for Mealtime Champions (and a dining team) so that home areas can utilize implementation methods and tools that work best for their home area.	• Greater team engagement and self-reflection checklists mentioned above to bring awareness as to what aspects of mealtimes could be improved. • CHOICE Dining Team to include residents, family, care team champions, management and CHOICE Coach; ownership and leadership transferred from Coach to home members over the course of the implementation. • Training components for the CHOICE Dining Team on change management; mentorship by Coach on change management principles.
Keeping communication lines open	• More frequent communication was needed between the CHOICE Coach, the Mealtime Champions, and the home area for guidance and support in determining what aspects of mealtimes to target and how to go about it. • Once implementation is underway, clear and consistent methods of communication are needed to understand how the intervention is progressing and what support is needed in the home area.	• Establish communication processes with Choice Dining Team and greater team, including residents and family. • Use a variety of communication formats to reach diverse audiences and once established use consistently.
Sharing responsibilities and accountability	• The CHOICE Program gave the home areas the opportunity and space to reflect on how mealtimes could be improved and what their roles were to make changes. • Mealtime Champions expressed additional pressure associated with the responsibility of leading the change efforts in their home areas. • The development of a Dining Team would assist in sharing some of the change management tasks that were originally given to the Mealtime Champions.	• Development of a CHOICE Dining Team to share responsibility and promote accountability. • Use of informal audit of new practices to promote accountability and embedding.
Empowering and supporting creativity	• From the outset of implementation, care staff and management need to collaborate to identify feasible mealtime improvements and change management strategies that work best for their home area. • Care staff have creative ideas and solutions to improve the mealtime experience for residents, however, they require time, resources, and support to make them a reality.	• CHOICE Dining Team needs to include a management representative to facilitate some changes considered a priority. • To ensure that care staff do not feel overwhelmed with change efforts, 1–2 CHOICE Principles could be prioritized. • Provide opportunities for engaging staff in identifying solutions; potentially post review of evaluation data to stimulate priority setting and motivation for change.

#### New appreciation for mealtimes: “It’s not about us, it’s about them”

After implementation of the CHOICE Program, all participants expressed a new appreciation and greater understanding of the importance of the mealtime experience for residents in their home areas, *“I’m going to be honest, we forget that we are in* *their* *dining room” (PSW 2)*. Leadership saw CHOICE as “*a great opportunity for [them] to take a look at [the dining experience] more in-depth.”* (MG 2), but as demonstrated in both qualitative and quantitative data, this was only a start to making change within each of the home areas. The program also served as a way to reflect on how team members’ behaviours impacted residents’ mealtimes, *“I feel like it was a really big eye-opener about things we could change and how me and [the other team members] were acting in the dining room. These are human beings – they’re not robots. This is their home and we should be engaging with them” (PSW 3)*.

#### Knowing the context and culture

Dining rooms in Wellesley and Parker differed in terms of context and subculture, which meant that strategies to make change that worked well in one home area did not necessarily work well in the other. Gaining an understanding and appreciation for these differences meant tailoring how the CHOICE Principles were enacted to meet the mealtime needs of team members and residents. Characteristics of each home area (e.g., differences in the number of residents needing eating assistance) influenced which improvements could be easily adopted, and why others were not as acceptable; context drove what was done to improve dining and how it was implemented. As one RN explained, *“Wellesley is one of our heavier care units where we do struggle with team members a lot. I think that was a barrier in the research” (RN).* As there were more residents who required eating assistance on Wellesley, less team members were available to partake in mealtime processes such as serving food and socializing with residents.

#### Getting everyone on board

Interviews identified the importance of ensuring that care team members and management were aware of what would be involved in implementing the CHOICE Program from the outset. For example, team members felt that reassurance was needed in order to spend additional time socializing with residents without being penalized, *“You can’t sit down [at the dining table with residents], you’re going to get in trouble if the general manager walks on the floor and you’re sitting down” (PSW 1)*. Team members believed that CHOICE would give them the opportunity to dedicate their time and efforts to meals, *“I hear that a lot, ‘we don’t have time to get to talk with them or help them more than we want to’. So, this was actually the time when you can do it.” (DA 2).* However, dedicated time to the implementation process was identified by some leadership as difficult to achieve, *“I would have loved to have been more involved in it, but I found myself at times… I get pulled in every direction” (HM 1).* Restrictions in leadership’s ability to both dedicate time to the implementation process, as well as being aware of the additional time that team members require to execute CHOICE meal practices, may help to explain the lack of change in some aspects of the mealtime experience, such as a more relaxed dining environment, *“knowing that management is well aware that mealtime is going to take a bit longer” (PSW 3*).

#### Keeping communication lines open

Communication between the research team, team members, and leadership was needed in order to problem-solve and overcome barriers throughout the implementation process. In-person contact was made by the CHOICE Coach, MT Champion, and leadership on a bi-weekly basis; however, mechanisms that supported more frequent communication were needed between the two home areas. In response, the research team began teleconferences with MT Champions and leadership during those weeks where the CHOICE Coach was not on site. Selecting MT Champions who possessed effective communication skills and were respected by their co-workers was also vital to spreading the CHOICE Principles and components, *“to be honest, you picked a really good communicator… Communication, I think, is the biggest thing” (DA1).*

#### Sharing responsibilities and accountability

Team members and leadership expressed the need for accountability as a source of motivation for themselves to both critically evaluate current mealtime practices and to re-consider ways to improve mealtime experience for residents, *“With having you guys there, I noticed a lot of things that are wrong with our dining room service. The first time when you came, I thought, ‘Oh, we do everything great’. Well, these past couple of days I’ve noticed so many things that are wrong.” (DA2)*. Self-reflection and accountability to the research team and CHOICE Coach served as motivation for team members and leadership to focus on their weekly mealtime goals. Feeding back results from the mealtime audits specifically to champions and leadership also supported this accountability. Leadership recognized the need to be present in the home areas if the program was to be sustainable, *“We really need to make our spot checks, we need to ensure that what we’re saying is actually happening. Follow through and follow-up – that’s a huge component” (HM 2).*

#### Empowering and supporting creativity

Leadership and team members were the experts in knowing how CHOICE Principles could be enacted, what would be feasible and acceptable within the subculture and context of their home area. Part way through implementation and with the help of the CHOICE Coach they felt empowered to make the CHOICE Principles their own. Several team members took the initiative to implement creative ways to enhance the mealtime experience for residents, *“Having the ideas and trying them, and then once you’ve tried them it gives you the motivation to think of other things” (PSW 4)*. For instance, team members took it upon themselves to bake bread in the living room so as to entice residents’ appetites, which may have contributed to the significant improvement in the overall MTS physical environment score. Other team members held weekly Supper Clubs that involved a theme dinner with costumes, decorations, and music. These efforts were reflected in the MTS analysis where both the physical environment and overall mealtime ambiance scores improved over time. Ensuring that all team members felt that they had the capacity and opportunity to implement CHOICE was identified by team members as an area for improvement, in particular, the need for more reflection and hands-on approaches beyond education. In addition, targeting casual and part-time team members was identified as a priority to *“get them more comfortable with serving in the dining room, so that it’s not always the same people, so that they all get those skills and they’re more well-rounded” (DA 1)*.

## Discussion

Mealtimes are often the most anticipated daily event for residents living in LTC homes. However, opportunities for meaningful social engagement are oftentimes missed within these settings, where the communal dining room can result in the pleasurable aspects of a mealtime coming second to concerns surrounding efficiency and sufficient food intake [4, 32]. It is the authors’ contention that if mealtime experiences support relationships and positive social connections (a necessary aspect of quality of life), that improvements in nutritional intake will follow [11]. Team members and home management have an important role in assisting in defining and promoting a culture of care that centers around relationships, as it is this culture that ultimately impacts how residents experience their mealtimes [33].

This mixed methods developmental evaluation looked to determine whether the CHOICE Program was an intervention that was worthy of further development and testing. Despite each home identifying different areas for improvement, meta-inferences from both the quantitative and qualitative phases showed that the CHOICE Principles resonated with team members and were seen as a goal for dining. Those who were involved believed that the program was useful for addressing some of the challenges associated with mealtimes in their home areas [34]. Team members and management expressed a re-kindled appreciation for mealtimes and noted visible improvements in mealtime experiences in both home areas.

While the CHOICE Principles resonated with team members as a way to improve mealtimes, the feasibility of implementing and carrying out ways in which the principles could be enacted proved to be more challenging than initially anticipated. For example, as residents on Wellesley needed more one-on-one assistance with eating, there were fewer team members available to walk around the dining room to encourage conversation amongst residents and thus reach the goal of Connecting (refer to Table 1 for CHOICE Principles definitions). In this case, the motivation to perform certain aspects of the CHOICE Program on Wellesley was not the issue; it was a matter of addressing the feasibility of how that principle could be enacted within the context in order to create the opportunity for team members to engage in more socialization with residents. One strategy was inclusion of a recreation therapist at meals to support the care team. Contextual differences between home areas, played an important role in shaping how the CHOICE Principles were interpreted and enacted during meals; this confirms that linear, structured approaches to care within the LTC setting [4, 35] is a barrier to improvements that are contextually driven. When looking to build upon this developmental evaluation, there is a need to be responsive to influencing factors at the management and home levels that may hinder or promote the uptake of change initiatives early on in the implementation process [36, 37].

In addition, differences in improvements between the two home areas and feedback from interview participants reveal that more time with the Coach would have been beneficial to build capacity for both teamwork and change management techniques before change efforts were undertaken. No doubt, the large geographic distance (280 km) between the research team and the LTC resulted in less face-to-face time than what may have been needed with the home. This made it difficult to meet the needs of some team members and MT Champions during especially challenging times in the implementation process. Modifications to the CHOICE Program should acknowledge the face-time needed by the Coach [38] in order to empower team members, specifically MT Champions [39], so that they feel confident in applying change management approaches when tailoring principles in their home areas [40, 41]. Table 6 outlines key learnings and outcomes based on findings from this study, in addition to planned improvements for the future development of the CHOICE Program.

### Working to make mealtimes more relationship-centred

Results from this developmental evaluation demonstrate the potential for the CHOICE Program to improve some aspects of the mealtime experience, namely physical and overall quality of the dining environment. It is important to note however, that these changes were not necessarily experienced in both home areas to the same extent. For example, although there was a significant time by dining room change noted in the social summative scale, there was a decrease in performance for one area (Wellesley = 5.4 to 5.2) over time. From baseline, one home area improved the overall quality of the environment significantly more than the other, demonstrating that comparisons across neighbourhoods can be challenging, as they are not equivalent with respect to their capacity for improvement. In areas where residents have significant dementia, social interactions are likely to never be high due to their limited verbal capacity. Relationship-centred care may also be challenging due to the higher demands for hands-on care. The physical and overall quality of the mealtime experience, which significantly improved in both areas included in this pilot, appear to be more readily changeable with the CHOICE program. These findings not only reinforce the need to account for contextual differences between environments, but also to temper expectations as to what can realistically be achieved within a short-period of time based on staffing and team member skills as well as the capacity of residents. Making improvements to an organization’s care culture and social environment has been noted to be a challenging task [42] that requires additional time and effort when compared to making improvements to the physical dining environment [11]. Often such cultural changes require targeting roles and responsibilities with added quality supervision on the part of home leadership [36]. Thus, it is recommended that the feasible changes to the built environment be targeted in the initial stages of the implementation process, as altering the physical dining environment (i.e., clock on wall, decorations on table, music playing) provides visible improvements that change-making is underway.

### Study limitations

Future evaluation of CHOICE should consider the use of a wait list control through a stepped-wedge design rather than a randomized control trial, as this pilot clearly demonstrated that the context of units greatly influences uptake and discernible change. Methodological limitations of this study include the MTS potentially being too insensitive, with too few observations, to capture the gradual improvements that team members and leadership referred to during interviews [25]. The issue of social desirability from interview participants may have led to responses, which may not have been entirely reflective of their experiences implementing the program. As well, there is the potential for confirmation bias, as the interventionist was the interviewer. Other forms of quantitative assessment to capture improvements should include team members’ perspectives on mealtimes and dyads of the resident and family members. Furthermore, it should be noted that because of an illness outbreak at the LTC home, we were unable to capture the last point of data collection at 32 weeks, which may have indicated further improvements in the dining environment. Staff turnover was a barrier to continuity with initial study participants; most notably, a key individual from the home’s management team was unable to participate in the interview process due to extenuating circumstances, which would have undoubtedly revealed critical insights into the feasibility and acceptability of the program. The research team appreciates the importance of the inclusion of family and residents in a relationship-centred intervention, however, these perspectives were not attained in this evaluation. Future development of the CHOICE Program will extend qualitative data collection to family and residents as well.

## Conclusions

The CHOICE Program demonstrated that it was possible to improve some aspects of the mealtime experience in two home areas within a relatively short period of time. Capacity for improvements in relationship-centred and social interactions appear to be more challenging and less consistent, as they are likely influenced by resident care needs (e.g. need for eating assistance). CHOICE gave team members and home management the opportunity to (re)consider the importance of mealtimes for residents living in LTC homes. Tailoring program components and implementation methods is absolutely critical for initial uptake and sustainability of change efforts. This involves including not only team members in the implementation of CHOICE, but also a vested interest and support from home management. Future directions for CHOICE development include a more collaborative approach to tailoring, implementation and evaluation, including the involvement of residents, family members, and volunteers, as they have the capacity to enhance and support relationship-centred dining.

## Additional file

Additional file 1: **Table S1.** "Description and Linear Mixed Model Analysis of MTS Subscales At Each Time Point By Home Area”. This table provides the descriptive analysis of the six MTS subscales for each of the home areas over time. The table also provides results of the linear mixed model analysis of each of these MTS subscales for both home areas by dining room, time, and the interaction between dining room and time. (DOCX 23 kb)

## Abbreviations

ADL-LF: Minimum Data Set Activities of Daily Living-Long Form
CPS: Cognitive Performance Scale
DA: Dietary Aid
DFS: Director of Food Service
HM: Home Management
LTC: Long-Term Care
MDS: Minimum Data Set
M-RCC: Mealtime Relational Care Checklist
MT Champions: Mealtime Champions
MTS: Mealtime Scan for Long-Term Care
PSW: Personal Support Worker
RCC: Relationship-Centred Care
RIA: Research Institute for Aging
RN: Registered Nurse
RPN: Registered Practical Nurse
