# Supplementary material for: Mixed methods developmental evaluation of the CHOICE program: a relationship-centred mealtime intervention for long-term care
Source: BMC Geriatr. 2018 Nov 13;18:277. doi: 10.1186/s12877-018-0964-3 (PMC6234643; doi:10.1186/s12877-018-0964-3)
Supplement: Supplementary file 1 — Table S1. "Description and Linear Mixed Model Analysis of MTS Subscales At Each Time Point By Home Area”. This table provides the descriptive analysis of the six MTS subscales for each of the home areas over time. The table also provides results of the linear mixed model analysis of each of these MTS subscales for both home areas by dining room, time, and the interaction between dining room and time. (DOCX 23 kb) [file 12877_2018_964_MOESM1_ESM.docx]

***Table S1.*** Descriptives and Linear Mixed Model Analysis of MTS Subscales At Each Time Point By Home Area

|  | **Descriptives by dining room** | | **Mixed model analysis with interaction** | |
| --- | --- | --- | --- | --- |
|  | **Summative Scale Scores, Mean (SD)^a^** | | **Effect** | **p-value^b^** |
| **Time Point (weeks)** | **Wellesley** | **Parker** |  |  |
|  | **Orientation Cues, [0-14]^c,d^** | | **Orientation Cues, [0-14]^c,d^** | |
| 0 | 5.8 (1.92) | 5.6 (1.52) | Dining Room | 0.04 |
| 8 | 5.6 (1.82) | 6.4 (1.14) | Time | 0.13 |
| 16 | 5.4 (2.30) | 6.0 (0.71) | Dining Room x Time | 0.12 |
| 24 | 5.6 (1.82) | 8.8 (0.84) |  |  |
|  | **Excess noise, [0-27]^e^** | | **Excess noise, [0-27]^e^** | |
| 0 | 4.0 (3.08) | 4.0 (2.00) | Dining Room | <0.01 |
| 8 | 1.6 (0.55) | 5.8 (2.28) | Time | 0.37 |
| 16 | 3.4 (1.34) | 5.2 (2.28) | Dining Room x Time | 0.09 |
| 24 | 1.0 (1.00) | 4.6 (1.95) |  |  |
|  | **Social interaction, [0-36]^d,f^** | | **Social interaction, [0-36]^d,f^** | |
| 0 | 12.2 (1.30) | 15.4 (6.54) | Dining Room | <0.01 |
| 8 | 10.8 (2.39) | 17.0 (7.24) | Time | 0.29 |
| 16 | 8.6 (1.95) | 17.0 (5.83) | Dining Room x Time | 0.62 |
| 24 | 12.8 (4.15) | 21.0 (5.96) |  |  |
|  | **Task focused interaction, [0-32]^e,g^** | | **Task focused interaction, [0-32]^e,g^** | |
| 0 | 6.4 (1.14) | 9.0 (3.24) | Dining Room | <0.01 |
| 8 | 6.0 (1.22) | 10.0 (3.32) | Time | 0.31 |
| 16 | 4.0 (2.34) | 8.6 (2.41) | Dining Room x Time | 0.85 |
| 24 | 4.2 (3.03) | 8.2 (3.27) |  |  |
|  | **M-RCC, [0-68]^d^** | | **M-RCC, [0-68]^d^** | |
| 0 | 37.3 (4.68) | 38.2 (3.35) | Dining Room | 0.15 |
| 8 | 39.0 (2.85) | 35.9 (1.39) | Time | <0.01 |
| 16 | 36.1 (5.02) | 39.8 (1.96) | Dining Room x Time | 0.08 |
| 24 | 40.8 (5.93) | 46.4 (2.70) |  |  |
|  | **RCC when assisting with eating, [0-36]^d^** | | **RCC when assisting with eating, [0-36]^d^** | |
| 0 | 16.8 (4.01) | 23.2 (1.92) | Dining Room | 0.05 |
| 8 | 20.8 (2.61) | 20.1 (2.72) | Time | 0.88 |
| 16 | 20.5 (6.73) | 22.0 (1.94) | Dining Room x Time | 0.15 |
| 24 | 19.7 (3.15) | 21.3 (1.68) |  |  |

^a^n=5 observations per time point per dining room

^b^P-values from type 3 test of fixed effects

#### ^c^List of potential orientation cues includes food aroma, decorations on table, meal menu on table, table setting, tablecloth, condiments, contrast between plate and food

#### ^d^Values in square brackets indicate range of possible values; a higher score is better

^e^­­Values in square brackets indicate range of possible values; a lower score is better

#### ^f^Social interaction = sum of all of the positive social interactions.

^g^Task-focused interaction = sum of all task-focused interactions.

Abbreviations: RCC= relationship-centred care; SD= standard deviation*.*
